# Phylogenetic and Molecular Profile of *Staphylococcus aureus* Isolated from Bloodstream Infections in Northeast Brazil

**DOI:** 10.3390/microorganisms7070210

**Published:** 2019-07-22

**Authors:** Andrea de S. Monteiro, Bruna L. S. Pinto, Joveliane de M. Monteiro, Rômulo M. Ferreira, Patrícia C. S. Ribeiro, Silvia Y. Bando, Sirlei G. Marques, Luís C. N. Silva, Wallace R. Nunes Neto, Gabriella F. Ferreira, Maria Rosa Q. Bomfim, Afonso G. Abreu

**Affiliations:** 1 Programa de Pós-Graduação, Universidade Ceuma, São Luís 65075-120, Brazil; 2 Laboratório Cedro, São Luís 65020-570, Brazil; 3 Departamento de Pediatria, Faculdade de Medicina da Universidade de São Paulo (FMUSP), São Paulo 01246-903, Brazil; 4 Hospital Universitário da Universidade Federal do Maranhão, São Luís 65020-070, Brazil; 5 Departamento de Farmácia, Universidade Federal de Juiz de Fora, Governador Valadares 35010-177, Brazil; 6 Programa de Pós-Graduação em Ciências da Saúde, Universidade Federal do Maranhão, São Luís 65080-805, Brazil

**Keywords:** *Staphylococcus aureus*, bloodstream infection, multilocus sequence typing (MLST), antimicrobial resistance

## Abstract

*Staphylococcus aureus* is a notorious human pathogen associated with serious nosocomial and community-acquired infections, such as pneumonia, meningitis, endocarditis, toxic shock syndrome, and sepsis, among others. The objective of this study was to investigate the molecular profile, antimicrobial resistance, and clonal diversity of *S. aureus* isolated from the bloodstream. The determination of the minimum inhibitory concentration (MIC) of the antimicrobial was performed by an automated method. The presence of several virulence and resistance genes was evaluated by PCR. In addition, multilocus sequence typing (MLST) was used to analyze the clonal diversity of *S. aureus*. A high resistance to oxacillin (78%), clindamycin (78%), erythromycin (70%), ciprofloxacin (61%), and gentamicin (52%) was observed among the isolates. In most of them, the following virulence genes were detected: *hlb* (83%), *ebpS* (61%), *icaA* (57%), *fnbpA* (17%), and *clfA* (13%). Only one isolate carried the *pvl* gene. MLST analysis identified five new sequence types (STs): 5429, 5430, 5431, 5432, and 5433, as well as another seven—ST5, ST97, ST398, ST101, ST30, ST461, and ST2779—among the remaining strains. These seven STs and the four new STs are clustered in four clonal complexes: CC1, CC2, CC7, and CC17. Phylogenetic analysis showed the genetic relationship of the five new ST strains with another 18 strains. Altogether, these analyses indicate the horizontal transfer acquisition of virulence factor genes and multidrug resistance.

## 1. Introduction

Nosocomial infections remain among the leading causes of mortality worldwide, with millions of deaths globally each year. These clinical conditions result in high economic and social costs for society. The infection severity is increased by some factors, such as the immunocompromised state of the patient and the presence of bacteria strains with a multidrug-resistance profile [1,2,3,4]. The use of medical devices is also considered an important risk factor for the development of bloodstream infections that are the leading cause of death for hospitalized patients [5].

*Staphylococcus aureus* is a frequent agent of potentially fatal bloodstream infections, especially due to its remarkable ability to colonize different superficies (including medical devices) and to spread in several environments. The estimated incidence of *S. aureus*-induced bloodstream infections is around 80 to 190 cases/100,000 inhabitants per year in developed countries [6,7,8]. The development of several mechanisms of virulence by *S. aureus* has ensured its success during systemic infections, which include factors involved in human immune system evasion, surface proteins, secreted enzymes, and toxins that damage the host membranes [9,10].

From the repertoire of enzymes for virulence, hemolysins produced by *S. aureus* are an important factor, with cytotoxic action responsible for lysing erythrocytes and culminating in a worsening of clinical symptoms during bloodstream infections [11]. Other toxins like those forming pores in leukocytes, such as Panton Valentine leukocidin (PVL), may aggravate pulmonary infections in episodes of necrotizing pneumonia [12]. In addition, *S. aureus* also has the ability to utilize fibronectin-binding protein A (FnBPA) to adhere to and consequently be internalized into host cells. This results in longer persistence in the tissues, as well as evasion from the attack of the immune system [13]. Another important virulence factor is elastin-binding protein (EbpS), a microbial surface component recognizing the adhesive matrix molecule that mediates bacterial cell binding to soluble elastin and tropoelastin [14].

Furthermore, *S. aureus* has acquired great medical importance due its ability to become resistant to multiple antimicrobial drugs through different molecular pathways [15]. The classical example is the emergence of methicillin-resistant *S. aureus* (MRSA) strains, which raises great concerns worldwide. MRSA is estimated to cause millions of deaths per year in the US [16,17], and the prevalence of MRSA in Latin America appears to be very heterogeneous, ranging from 6% in Central America to 80% in some South American countries, including Brazil [8,18,19]. The β-lactam resistance in *S. aureus* is primarily due to expression of the *mecA* gene, which is present on the chromosome in a resistance cassette, called Staphylococcal Cassette Chromosome (SCC) *mec*, which encodes the low affinity penicillin-binding protein (PBP2a) [20]. Besides MRSA, other strains of *S. aureus* have been detected as multidrug-resistant (*MDR)* and showing resistance towards last-resort drugs such as vancomycin (VRSA) and linezolid [21,22].

Therefore, the early detection of the arsenal of virulence and drug resistance genes in *S. aureus* is an important task in order to efficiently track the MDR and hypervirulent strains associated with high levels of morbidity/mortality. The purpose of the present study was to investigate the genetic diversity and antimicrobial susceptibility of *S. aureus* isolated from bloodstream infections in patients hospitalized in Intensive Care Units (ICUs) in different public hospitals in São Luis (Maranhão state, Northeast Brazil), as well as to compare the phylogenetic profile among the isolates.

## 2. Material and Methods

### 2.1. Bacterial Strains

*S. aureus* analyzed in this study were obtained from positive blood cultures after incubation in Bactec Plus Aerobic/F and Bactec Lytic/10Anaerobic/F blood culture bottles (Becton Dickinson, Sparks, MD, USA). The blood samples were collected from patients treated at different public hospitals in São Luis, Northeast Brazil, during a period of seven months. The isolation from positive blood cultures was performed using blood agar plates (BioMérieux, Marcy l’Etoile, France). Subsequently, the isolates were identified by MALDI-TOF (Matrix Assisted Laser-Desorption Ionization-Time of Flight) Mass Spectrometry (MS). The mass spectra acquired for each bacterial strain were compared to the mass spectra contained in the database using Biotyper 3.0 software (Bruker, Billerica, MA, USA). All isolates were kept in Luria-Bertani broth supplemented with 15% glycerol at −80 °C.

### 2.2. Antimicrobial Susceptibility Testing

The antimicrobial susceptibility profile of each *S. aureus* isolate was determined using AST#105 and GP-ID cards by the VITEK® 2 Compact system (BioMérieux, Marcy l’Etoile, France), according to the Clinical Laboratory Standards Institute (CLSI, 2016). All *S. aureus* isolates were tested for their susceptibility to the following antibiotics: oxacillin, erythromycin, clindamycin, gentamicin, rifampicin, teicoplanin, vancomycin, trimethoprim/sulfamethoxazole, ciprofloxacin, and linezolid.

### 2.3. Analysis of Virulence and Resistance Genes

The presence of several virulence (*clfA*, *cna*, *ebpS*, *eta*, *etb*, *fnbpA*, *hla*, *hlb*, *hlg*, *icaA*, *pvl*, *sdrE*, *sea*, *seb*, *sec*, *sed*, *see*, *seg*, *seh*, *sei*, *sej*, *sirB*) and resistance genes (*blaZ* and *mecA*) was assessed by PCR. Primer sequences, sizes of amplified products, and annealing temperatures are described in Table 1. Amplification reactions were performed in a total volume of 25 μL containing 1 U reaction buffer of GoTaq Green Master Mix (Promega Corporation, Madison, USA), 0.25 μM of each primer and 20 ng of template DNA extracted.

### 2.4. DNA Sequencing and Multilocus Sequence Typing (MLST)

Double-stranded DNA was obtained using the Gentra Puregene kit (Qiagen, Hilden, Germany). The sequencing of the 16S rRNA nucleotide sequence and the fragments of seven housekeeping gene amplicons was performed in the Illumina platform (Ilumina Inc., San Diego, CA, USA). Reactions were performed according to the manufacturer’s instructions, using the MiSeq V250 kit (Illumina Inc., San Diego, CA, USA). The primers used for the PCR amplifications were previously described [26,27]. The 16S rRNA nucleotide sequence was compared with sequences in the GeneBank database, using BLASTn software to confirm the identification of the species.

Multilocus sequence typing (MLST) analysis (based on seven housekeeping genes: *arcC*, *aroE*, *glpF*, *gmk*, *pta*, *tpi*, and *yqiL*) was used for the identification of sequence type (ST) and clonal complex (CC). The STs were obtained through an online webtool (http://saureus.mlst.net), and the CCs were identified by eBURSTv3 software (http://eburst.mlst.net/). Moreover, the eBURST groupings were obtained using the most conservative group identification of related STs, i.e., all members in the same group share identical alleles—at least six out of seven loci—with at least one other member of the group. Additionally, we conducted a phylogenetic analysis using all seven housekeeping gene fragments and the 16S rRNA nucleotide sequence. Phylogeny was inferred using the Neighbor-Joining method [28], and genetic distances were obtained by the Kimura 2-parameter method [29]. The phylogeny construction was conducted in MEGA6 software [30].

Nucleotide sequence alignment analysis was accomplished in BioEdit Sequence Alignment Editor software (version 7.0.5), using ClustalW (University College Dublin, Dublin, Ireland). The comparative analysis was performed using a consensus sequence constructed with all 675 sequences deposited in the MLST database.

### 2.5. Ethical Aspects

This research was approved by the Research Ethics Committee of CEUMA University (protocol n° 813.293/2014).

## 3. Results

### 3.1. Staphyloccocus spp. Strains Presented a High Resistance Profile Towards the Tested Antimicrobials

In this study, a total of 23,718 aerobic blood cultures from patients at different public hospitals were analyzed. Among them, 91 *Staphylococcus* spp. were identified: *S. aureus* (35 isolates), *S. epidermidis* (22), *S. haemolyticus* (21), *S. hominis* (6), *S. capitis* (2), *S. warneri* (2), *S. caprae* (1), *S. saprophyticus* (1), and *S. cohnii* (1). For the characterization and phylogenetic analyses, a total of 23 *S. aureus* were randomly selected. Most of them were resistant to oxacillin (78%), clindamycin (78%), erythromycin (70%), ciprofloxacin (61%), and gentamicin (52%), and all isolates showed sensitivity to vancomycin and linezolid (Table 2).

### 3.2. S. aureus Strains Isolated from Blood Infections Displayed a Diversity of Virulence and Resistance Genes

Given the high levels of drug resistance in *S. aureus*, the presence of several genes associated with virulence and resistance pathways was investigated. A total of 8 strains (35%) were positive for the *mecA* gene, while 11 isolates (48%) harbored *blaZ*. Only three strains carried both *mecA* and *blaZ* genes (strains 1503, 1505, 1522). Regarding the virulence genes, the most prevalent was *hlb* (83%), followed by *ebpS* (61%), *icaA* (57%), *fnbpA* (17%), *clfA* (13%), and *pvl* (4%) (Table 2). However, none of these isolates harbored the *tsst*, *sea*, *seb*, *sec*, *sed*, *sec*, *see*, *seh*, *sej*, *eta*, or *etb* gene.

The evaluation of gene combinations revealed that most of the *S. aureus* isolates (52%) harbored both *hlb* and *ebpS* genes, and some isolates harbored them in combination with others: *icaA*, *blaZ*, *fnbpA*, and *mecA*. Particularly, *S. aureus* 1507 presented a high variety of resistance and virulence genes: *hlb*, *ebpS*, *icaA*, *blaZ*, *fnbpA*, *mecA*, *hlb*, *hlg*, as well as the *pvl* gene (Table 2).

### 3.3. MLST Analysis

MLST analysis identified seven STs among 18 isolates. Most of them belonged to ST5 (38%), followed by ST97 (12%), ST398 (4%), ST101 (4%), ST30 (4%), ST461 (4%), and ST2779 (4%). Five isolates (strains 1506, 1512, 1514, 1516, and 1519) had unknown *yqiL* nucleotide sequences, did not belonging to any ST. Therefore, the multiple alignment analysis of these five strains and the consensus sequence based on all sequence types were deposited in the MLST database, and the new STs were classified: 5429, 5430, 5431, 5432, and 5433, respectively to the five isolates.

**Figure 1:**
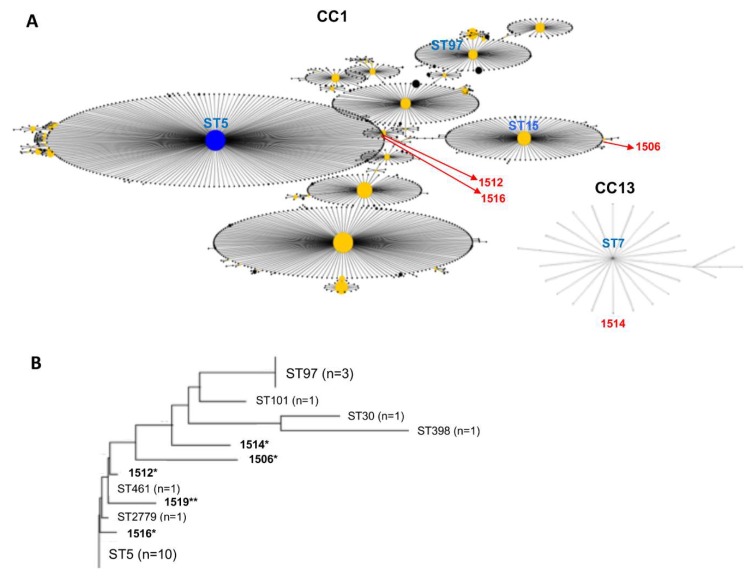
Clonal complex eBURST group analysis and the genetic relationship of the 23 *S. aureus* isolates. (**A**) The eBURST group was obtained for the four strains 1506, 1512, 1514, and 1516, based on six identical alleles (except for *yqiL*, four new sequences). The size of the circles shows the number of isolates for each ST (node), and the central node indicates the most prevalent ST in the CC group. Blue or yellow nodes indicate ST group founder or ST subgroup founder, respectively. The black line indicates one different allele between two ST groups. (**B**) Phylogeny shows the genetic relationship of strain 1519. * Indicates the strains also analyzed by eBURST and ** indicates the strain from which CC was inferred by phylogenetic analysis. A number between parentheses corresponds to the number of isolates.

The eBURST analysis identified 18 strains belonging to CC1 (ST5, ST97, ST461, and ST2779), CC2 (ST30), CC7 (ST398), and CC17 (ST101). This analysis was conducted for the five strains (1506, 1512, 1514, and 1516) using six alleles (except for *yqiL*) to identify the most related STs and the corresponding CCs. Strain 1519 has a sequence type 631 for *arcC* and, based on six alleles, has ST5286, and has a new *yqiL* sequence, thus the CC could not be identified. The classification of this strain on CC1 was inferred by phylogenetic analysis (Figure 1). Regarding the three strains 1512, 1516, and 1506, belonging to CC1, two of them, 1512 and 1516, have six identical alleles with the ST5 group. The other strain 1506 has six identical alleles with the ST15 group. Strain 1514 has six identical alleles with the ST7 group of the CC13 (Table 2, Figure 1A). Additionally, we constructed a phylogeny of all 23 strains (Figure 1B). Phylogenetic analysis showed that strain 1519 is closely related with ST461 (strain 1509) and ST2779 (strain 1502), belonging to the CC1 group.

## 4. Discussion

The emergence and spread of pathogens, such as *S. aureus*, is an important public health concern, making a better characterization of the virulence and drug resistance profiles of these strains of vital importance. In this study, we employed different approaches to evaluate the genetic diversity of *S. aureus* strains isolated from bloodstream infections in patients at several hospitals in São Luís (Northeast Brazil).

Among the *S. aureus* strains evaluated in this study, about 78% were MRSA. The isolation of MRSA has been common worldwide. This pathogen is one of the leading causes of nosocomial infections [31], representing 63% of staphylococcal infections [8,19]. In Brazil, several studies have demonstrated a prevalence of hospital-acquired *S. aureus* infections varying from 17% to 26%, and, approximately 70% to 100% of these infections are caused by multidrug-resistant strains [8,18,32].

In particular, a previous study performed in this same area using strains isolated from different samples of patients reported a prevalence of 29.4% for MRSA [32]. Therefore, our study indicates that there was a drastic increase in MRSA over the last decade. This phenomenon has also been observed in other studies, and it has been associated with different aspects such as the indiscriminate use of antibiotics in different contexts, socio-economic and environmental factors, migrations and hygienic conditions [8,18,33,34]. Increased bacterial resistance towards several antimicrobials makes vancomycin the drug of choice for the treatment of *S. aureus* MDR strains. Strains with resistance to vancomycin or linezolid were not observed in this study; however, VRSA isolates have been reported worldwide, including in Brazil since December 2012 [35]. In this way, the importance of analyzing the sensitivity profile of microorganisms to antimicrobial drugs is emphasized, because it is then possible to understand how they are distributed and how the choice of antibiotics should be made [36].

Regarding the detection of virulence factors, the *hlb* gene (which encodes hemolysin B) was detected in 83% of the *S. aureus* strains. Β-hemolysin has both sphingomyelinase and DNA biofilm ligase activities, and it can lysis a range of host cell membranes. This cytotoxin is very prevalent in *S. aureus* strains, and, although its exact contribution to *S. aureus* virulence remains unsolved, this toxin has been associated with cases of furunculosis, endocarditis chronic osteomyelitis, and respiratory infections in humans, and with mastitis in animals. The toxin also enhances the activity of phenol-soluble modulin, and its activity is enhanced by superantigens [27,37,38,39].

Other genes, such as *ebpS*, *icaA*, *fnbpA*, *clfA*, and *pvl*, were also detected in both MRSA and methicillin-sensitive isolates (MSSA). However, the gene encoding PVL was only identified in one MRSA isolate. This strain also showed the higher number of virulence genes. Most isolates carrying the *pvl* gene are MRSA, and a report showed an increase in *pvl*-positive strains in the period 2005–2009, where the minority of the isolates were methicillin-sensitive [40]. PVL confers increased virulence to *S. aureus* strains and it is believed that this toxin is a key factor in pneumonia pathogenesis. Furthermore, it induces toxic effects in neutrophils, intensifying the damage related to necrotizing pneumonia in humans [41,42].

Besides that, we investigated the presence of genes related to tissue adhesion and encoding proteins present on the cell surface. The results showed that *ebpS* (encoding the elastin-binding protein) and *fnbpA* (encoding fibronectin-binding protein A) genes were present in 61% and 17% of the *S. aureus*, respectively. Both *fnbpA* and *ebpS* appear to be widely distributed among *S. aureus* in hospitalized patients, and the frequency of these genes has varied in accordance with the type of sample and area studied [43,44,45,46,47]. It is worth mentioning that the binding of EbpS (encoded by *ebpS*) with elastin may promote bacterial colonization to facilitate pathogenesis [48]. In addition, the *fnbpA* gene is responsible for the expression of host adhesion and invasion proteins, and may be related to biofilm formation. Thus, FnbpA encoded by *fnbpA* is a very relevant factor for the action of bacterial pathogens in humans [49].

It is also worth highlighting the high prevalence of *icaA* (57%) in these strains; this gene was present in almost all MRSA strains. Various studies have also reported the high prevalence of MRSA carrying *icaA* [44,47]. This gene is associated with biofilm formation, a well-known risk factor for chronic infection, which constitutes a suitable environmental for the horizontal transfer of genes [50,51,52,53].

The ability to identify *S. aureus* correctly is important for understanding it and for making public health decisions. *S. aureus* has a diverse clonal population and studies have shown, through the MLST technique, that a small set of clonal complexes are associated with MRSA epidemics [54,55,56]. In addition, MLST has been used to characterize and investigate the distribution of *S. aureus* clones associated with human infections [8,57,58,59,60,61,62].

Based on the frequency of the virulence and antibiotic resistance genes (ARGs) found in this study, some housekeeping genes were sequenced for phylogenetic evaluation. This analysis allowed the distribution of these *S. aureus* isolated from bloodstream infections in five clonal complexes and seven STs. In these, the ST5 clone was the most frequent in our study, a result that corroborates those found in a prospective cohort multicenter study of *S. aureus* bacteremia conducted in Latin America in 2011–2014 [8] and Minnesota (USA) in 2015 [61]. ST5 was also the most prevalent in a recent study carried in China [46]. There is evidence to support that the ST5 clone has spread from humans to poultry [63] and is also present in dairy environments in the USA [64], in the Brazilian dairy industry [65], and in chickens [66].

It is known that clonal diversity in *S. aureus* differs according to the country or region, and that clones differ in antimicrobial resistance patterns and virulence factors [67]. In this study, we also showed a high frequency of the ST97 clone among the *S. aureus* isolates. This clone has been also recovered in the majority of isolated cases related with bovine mastitis [34,68]. In addition, the MRSA ST97 clone has been also described in pigs [69]. It is interesting to mention that these studies showed different species of animals sharing the same ST among them.

In this study, only one *S. aureus* isolate was classified as a ST398 clone. This ST398 of clonal lineage does not seem to be frequently represented among the *S. aureus* population [70]; however, it has been involved in sporadic cases of clinical disease with variable severity in humans [68,70,71,72,73,74,75]. The clonal complex 398 is among the most important livestock-associated *S. aureus* genotypes, and contact with animals is a well-described risk factor for infection in humans [60]. It has been found in pigs and in the nasal cavity of pig farmers in France [73,76], in swine herds [77], and in bovines [78].

In addition, one *S. aureus* strain belonging to the ST30 clone and carrying the *pvl* gene was detected in this study. Since the mid-1990s, MRSA ST30 clones with different types and genetic characteristics have been reported in many parts of the world [79]. These *S. aureus* strains are a major source of high-risk infection globally [80], and ST30 isolates carrying *pvl* are often associated with necrotizing pneumonia, which causes high morbidity and mortality rates in children [67,79,81].

The CC1 and CC2 groups seem to harbor most of the *S. aureus* isolates with multidrug resistance profiles. It is interesting to note that all four new STs (CC1 group) have multidrug resistance profiles (CIP, CLI, ERI, GEN, OXA, Table 2), and these STs are closely related. In addition, two strains of these new STs have many virulence genes (more than four). Moreover, the strains of ST5 and ST97 presented different virulence profiles; this result shows horizontal gene acquisition. It is noteworthy that *S. aureus* strains that are genetically closely related or that belong to the same ST were found in different hospitals, suggesting a spread of bacteria from the same clone in different environments. This is worrying since these bacteria can be disseminated from one hospital to another through the wrong medical and aseptic conduct of the health professional or through patient transfer between hospitals.

## 5. Conclusions

In summary, this study reports that *S. aureus* strains isolated from blood infections present a very high antimicrobial resistance profile, with ST5 and ST97 clones being the most frequent among *S. aureus* strains. In addition, it was possible to classify five new STs: 5429, 5430, 5431, 5432, and 5433. This population was mainly composed of MRSA isolates that presented a high and diverse frequency of virulence genes, representing a potential risk to public health. In addition, phylogenetic analysis can be useful for microbial characterization, knowledge, and the control of circulating bacterial clones in hospitals. Thus, the rapid identification of the causative agent of infection, antimicrobial susceptibility testing, and molecular techniques are very important in order to adjust the appropriate therapy and avoid ineffective treatments for microbial infections.

## Figures and Tables

**Table 1:** Primer used for PCR, size of amplified products, and annealing temperatures.

Gene	Primer Sequence (5′ - 3′)	Annealing Temperature (°C)	Size of PCR Product (bp)	Reference
Beta-lactamase (*blaZ*)	(F) ACTTCAACACCTGCTGCTTTC (R) TGACCACTTTTATCAGCAACC	61	173	[23]
Clumping factor A (*clfA*)	(F) GTAGGTACGTTAATCGGTT (R) CTCATCAGGTTGTTCAGG	50	1548	[23]
Collagen adhesin (*can*)	(F) AGTGGTTACTAATCATG (R) CAGGATAGATTGGTTTA	45	1722	[23]
Elastin-binding Protein (*ebpS*)	(F) CAATCGATAGACACAAATTC (R) CAGTTACATCATCATGTTTA	50	506	[23]
Exfoliative toxin A (*eta*)	(F) ACTGTAGGAGCTAGTGCATTTGT (R) TGGATACTTTTGTCTATCTTTTTCATCAAC	55	190	[24]
Exfoliative toxin B (*etb*)	(F) CAGATAAAGAGCTTTATACACACATTAC (R) AGTGAACTTATCTTTCTATTGAAAAACACTC	55	612	[24]
Fibronectin-binding protein A *(fnbpA*)	(F) CACAACCAGCAAATATAG (R) CTGTGTGGTAATCAATGTC	50	1226	[24]
Alpha-hemolysin (*hla*)	(F) CTGATTACTATCCAAGAAATTCGATTG (R) CTTTCCAGCCTACTTTTTTATCAGT	55	209	[24]
Beta-hemolysin (*hlb*)	(F) GTGCACTTACTGACAATAGTGC (R) GTTGATGAGTAGCTACCTTCAGT	55	309	[24]
Gama-hemolysin (*hlg*)	(F) GTCAAAGAGTCCATAATGCATTTAA (R) CACCAAATGTATAGCCTAAAGTG	55	535	[24]
Intracellular adhesion (*icaA*)	(F) GATTATGTAATGTGCTTGGA (R) ACTACTGCTGCGTTAATAAT	50	770	[23]
Methicillin resistance (*mecA*)	(F) GGTCCCATTAACTCTGAAG (R) AGTTCTGCAGTACCGGATTTTGC	57	163	[23]
Panton-valentine leucocidin (*pvl*)	(F) ATCAATAGGTAAAATGTCTGGACATGATCCA (R) GCATCAAATGTATTGGATAG AAAAGC	55	433	[25]
Serine-aspartate repeat-containing protein E (*sdrE*)	(F) CAGTAAATGTGTCAAAAGA (R) TTGACTACCAGCTATATC	50	749	[23]
Enterotoxin A (*sea*)	(F) GAAAAAAGTCTGAATTGCAGGGAACA (R) CAAATAAATCGTAATTAACCGAAGGTTC	55	560	[24]
Enterotoxin B *(seb*)	(F) ATTCTATTAAGGACACTAAGTTAGGGA (R) ATCCCGTTTCATAAGGCGAGT	55	404	[24]
Enterotoxin C (*sec*)	(F) GTAAAGTTACAGGTGGCAAAACTTG (R) CATATCATACCAAAAAGTATTGCCGT	55	297	[24]
Enterotoxin D (*sed*)	(F) GAATTAAGTAGTACCGCCCTAAATAATATG (R) GCTGTATTTTTCCTCCGAGAGT	55	492	[24]
Enterotoxin E (*see*)	(F) CAAAGAAATGCTTTAAGCAATCTTAGGC (R) CACCTTACCGCCAAAGCTC	55	482	[24]
Enterotoxin G (*seg*)	(F) AATTATGTGAATGCTCAACCCGATC (R) AAACTTATATGGAACAAAAGGTACTAGTTC	55	642	[24]
Enterotoxin H (*she*)	(F) CAATCACATCATATGCGAAAGCAG (R) CATCTACCCAAACATTAGCACC	55	376	[24]
Enterotoxin I (*sei*)	(F) CTCAAGGTGATATTGGTGTAGG (R) AAAAAACTTACAGGCAGTCCATCTC	55	576	[24]
Enterotoxin J (*sej*)	(F) TCAGAACTGTTGTTCCGCTAG (R) GAATTTTACCAYCAAAGGTAC	55	138	[24]
Siderophore compound transporter permease protein (*sirB*)	(F) CAGCTACGGCTACCGAAATA (R) CATTTTTGGGGGCTATTGTTGT	61	399	[23]

**Table 2:** Presence of virulence factor and antibiotic resistance profile of the 23 *S. aureus*, and their distribution in sequence type (ST) and clonal complex (CC). Blood samples were collected from patients treated at different public hospitals in São Luis, Northeast Brazil, and submitted to antimicrobial susceptibility testing, as well as to the detection of several virulence and resistance genes.

			Virulence Factor Profile										Antibiotics Resistance Profile							
CC	ST	Isolates	*blaZ*	*clfA*	*ebpS*	*fnbpA*	*hla*	*hlb*	*hlg*	*icaA*	*mecA*	*pvl*	CIP	CLI	ERI	GEN	OXA	RIF	TEI	TRI
CC1	97	1522	−	−	+	+	−	+	−	+	+	−	+	+	+	+	+	−	−	−
CC1	97	1523	−	−	−	−	−	+	−	+	+	−	−	−	−	−	−	+	−	−
CC1	97	1521	−	−	+	−	−	+	−	+	−	−	+	+	+	+	+	−	−	−
CC17	101	1500	−	−	−	−	+	−	−	−	−	−	−	−	+	−	−	−	−	−
CC2	30	1507	+	−	+	+	−	+	+	+	+	+	+	+	+	+	+	−	+	−
CC7	398	1520	−	−	−	−	−	+	−	+	−	−	−	+	−	−	+	−	−	−
CC13 *	5431	1514	−	−	−	−	−	+	−	−	−	−	−	+	−	−	+	−	−	−
CC1 *	5429	1506	−	−	−	−	+	+	−	−	−	−	+	+	+	+	+	−	−	−
CC1 *	5430	1512	−	−	+	+	−	+	−	+	+	−	+	+	+	+	+	−	−	−
CC1	461	1509	−	−	+	−	−	+	−	−	−	−	+	+	+	+	+	−	−	−
CC1 **	5433	1519	+	−	+	−	−	+	−	+	−	−	+	+	+	+	+	−	−	−
CC1	2779	1502	+	−	+	−	−	+	−	+	−	−	−	+	+	−	−	−	−	−
CC1 *	5432	1516	+	−	+	−	−	−	−	−	+	−	+	+	+	+	+	−	−	−
CC1	5	1524	−	−	−	−	−	+	−	+	+	−	−	−	+	−	−	−	−	−
CC1	5	1518	−	+	−	−	−	+	−	−	−	−	−	−	−	−	+	+	−	+
CC1	5	1517	+	+	+	−	−	+	−	+	−	−	+	+	+	+	+	−	−	−
CC1	5	1515	+	−	+	−	−	+	−	−	−	−	+	+	+	−	+	+	−	−
CC1	5	1501	+	−	+	+	−	+	−	+	−	−	−	+	−	−	−	−	−	−
CC1	5	1503	−	−	+	+	−	+	−	+	+	−	+	+	+	+	+	−	−	−
CC1	5	1505	+	−	−	+	−	−	−	+	+	−	+	+	+	+	+	−	−	−
CC1	5	1510	−	+	+	−	−	+	−	−	−	−	+	+	−	−	+	−	−	−
CC1	5	1511	−	−	−	−	−	+	−	−	−	−	+	+	+	+	+	−	−	−
CC1	5	1508	+	−	−	−	−	+	−	−	−	−	−	−	−	−	+	+	−	+
**Total**	**n** **%**	**23** **100%**	**11** **48%**	**13** **57%**	**14** **61%**	**4** **17%**	**2** **8%**	**19** **83%**	**1** **4%**	**3** **13%**	**8** **35%**	**1** **4%**	**14** **61%**	**18** **78%**	**16** **70%**	**12** **52%**	**18** **78%**	**4** **17%**	**1** **4%**	**2** **9%**

* CC based on 6 alleles; ** CC inferred by dendrogram / CIP: Ciprofloxacin; CLI: Clindamycin; ERI: Erythromycin; GEN: Gentamycin; OXA: Oxacillin; RIF: Rifampicin; TEI: Teicoplanin; TRI: Trimethoprim-Sulfamethoxazole / (+): resistant and (−): sensitive / CC: clonal complex; sequence type (ST).
